# Results of minimally-invasive aortic-valve replacement in octogenarians

**DOI:** 10.1186/1749-8090-10-S1-A293

**Published:** 2015-12-16

**Authors:** Sobaran Sharma, Joseph George, Yasir Ahmed, Umair Aslam, Pankaj Kumar

**Affiliations:** 1 Cardiothoracic Surgery, Morriston Hospital, Swansea SA6 6NL, UK

## Background/Introduction

Minimally-invasive aortic-valve replacement (mini-AVR) via J-sternotomy has been shown to reduce surgical morbidity. Little data is available on the outcomes of mini-AVR in the very elderly population.

## Aims/Objectives

We assessed the clinical outcomes of mini-AVR in all octogenarians undertaken at our centre.

## Method

A single consultant surgeon routinely undertook the minimally-invasive approach via J-sternotomy for all isolated first-time aortic valve replacements. Operative records and clinical outcomes of all patients who had undergone miniAVR in our centre between 2006-2015 were retrieved from the national cardiac surgery database. Patient demographics, premorbid status, operative details and outcomes were evaluated.

## Results

171 mini-AVRs were undertaken between 2006 and 2015, out of which 41 patients were aged 80 or above. Patient demographics were as follows: mean age 83.8 years (range 80-91, SD 2.934), female gender 63.4%, diabetes mellitus 9.8%, pulmonary disease 22.0%, LV function: <30% in 7.3%, 30-5% in 17.1% and >50% in 75.6%, logistic euroSCORE 13.3 (interquartile range 8.44 - 14.7, SD 9.04). Overall in-hospital and 30-day mortality was 2.4% (1/41), re-exploration rate was 0.0%, renal failure requiring dialysis 2.4% (1/41), permanent pacemaker 2.4% (1/41), CVA 0.0%, conversion to full sternotomy 0.0%.

## Discussion/Conclusion

Despite high logistic EuroSCOREs, we have shown excellent results in octogenarians by this approach. In this era of transcatheter aortic valve implantation, mini-AVR needs to be in the armamentarium of the surgical team.

